# Hospital Festivals and Meetings

**Published:** 1895-02-02

**Authors:** 


### HOSPITAL FESTIVALS AND MEETINGS.

### NORTH-EASTERN HOSPITAL FOR CHILDREN.

A special meeting was held at the Mansion House on Monday, January 28th, in aid of this hospital. The Lord Mayor presided, and called attention to the very great straits to which it is reduced at the present time, being in debt to the amount of £4,500. An urgent appeal to the public was necessary, and he hoped it would be heartily responded to.

Lady Jeune gave a moving address, asking all who were present to do what might be in their power to help a charity which was deserving of the utmost support, describing the difficulties of site and situation under which this particular hospital suffered, and giving her own experience of the work it accomplishes in one of the poorest and most crowded parts of London. There are no other children's hospitals nearer than Shadwell on the one side and Great Ormond Street on the other; its wards are crowded, and more room is needed. Lady Jeune spoke strongly of the crying needs of children, the children who would be the men and women of the future, and of the importance of securing to them health and strength, concluding by begging her hearers, by the memory of children they had known and loved, to come to the rescue of the North-Eastern Hospital at the present crisis. The resolution then proposed was as follows: "That the North-Eastern Hospital for Children is one of the best and most useful institutions of its kind in London, and that this meeting is of opinion that no better means could be found for charitable contributions than the repayment of its debt of £4,500, and the increase of its annual subscriptions by £500 a year."

Mr. Bousfield, Q.C., M.P., seconded the resolution, and said he had much pleasure, as one of the representatives in Parliament of a district served by the hospital, in speaking in its support. He explained that the hospital was designed when finished to contain 100 beds, at present there being only 58. For completing another wing a sum of £15,000 would be required, of which £5,000 was already in hand for that special purpose, the interest at present going to the funds of the hospital. The hospital dealt only with the children of the poor, 1,000 out-patients being treated a week, between the ages of two and twelve years. Year by year the income had been less adequate to meet the expenditure, and money had to be borrowed for current expenses, with the result that unless help came the hospital would be dragged every year further into the mire of bankruptcy. Mr. Bousfield spoke of the duty devolving upon those who were responsible for the present concentration of population in the East-end of London, a population composed of workers who ministered to the comforts of the dwellers in better and airier districts. He considered it would be a disgrace to London if this hospital were allowed to sink further into debt, and a duty to see that an almost unique institution such as the North-Eastern Hospital should be adequately kept up.

Afterwards Mrs. Arthur Stannard ("John Strange Winter") amused the audience with "a few stories," and Miss Fanny Kay gave a pianoforte solo, while contributions were being collected among the audience.

There can be only one thing needed to stir up a deep and lasting interest in such a hospital as the North-Eastern for Children, and that is a personal acquaintance with its needs and its work. If our readers will turn to The Hospital for May 6th, 1893, page 93, they will find there set forth a description of the institution which should lead them to see for themselves the work which is being done there day by day. No hospital in London is more needed than this unpretentious building, and yet none seems to meet with less of that financial help in which it is in such sore need. Hidden away in the depths of the East-end, unseen from the public thoroughfare, it is almost in danger of being forgotten, and yet should it cease to exist it would leave a gap which would be severely indeed felt by those who live within its sphere of labour.

We must draw special notice to the "Children's Association Fund" in connection with the hospital, by means of which opportunity is given to richer and happier children to help their poor little brothers and sisters in many small ways. To make the hospital and its needs better known, to do some little needlework for the sick children, or by sending old clothes or toys or odds and ends of any sort, addressed to Miss Curno, the Matron, North-Eastern Hospital for Children, Hackney Road, E.; and by regularly putting by one penny a week as a contribution for the children—these are the ways in which the members of the Children's Association can materially help. Bags and collecting cards will be sent at once upon application to the Hon. Secretary, Miss E. M. Lister, Leytonstone, Essex.

